# LoRa-Based Physical Layer Key Generation for Secure V2V/V2I Communications

**DOI:** 10.3390/s20030682

**Published:** 2020-01-26

**Authors:** Biao Han, Sirui Peng, Celimuge Wu, Xiaoyan Wang, Baosheng Wang

**Affiliations:** 1 School of Computer, National University of Defense Technology, Changsha 410073, China; pengsirui18@nudt.edu.cn (S.P.); wangbaosheng@126.com (B.W.); 2 Graduate School of Informatics and Engineering, The University of Electro-Communications, Tokyo 182-8585, Japan; celimuge@uec.ac.jp; 3 College of Engineering, Ibaraki University, Mito 310-8512, Japan

**Keywords:** V2V/V2I communications, automatic vehicle security, physical layer key generation, LoRa

## Abstract

In recent years, Vehicle-to-Vehicle (V2V) and Vehicle-to-Infrastructure (V2I) communication brings more and more attention from industry (e.g., Google and Uber) and government (e.g., United States Department of Transportation). These Vehicle-to-Everything (V2X) technologies are widely adopted in future autonomous vehicles. However, security issues have not been fully addressed in V2V and V2I systems, especially in key distribution and key management. The physical layer key generation, which exploits wireless channel reciprocity and randomness to generate secure keys, provides a feasible solution for secure V2V/V2I communication. It is lightweight, flexible, and dynamic. In this paper, the physical layer key generation is brought to the V2I and V2V scenarios. A LoRa-based physical key generation scheme is designed for securing V2V/V2I communications. The communication is based on Long Range (LoRa) protocol, which is able to measure Received Signal Strength Indicator (RSSI) in long-distance as consensus information to generate secure keys. The multi-bit quantization algorithm, with an improved Cascade key agreement protocol, generates secure binary bit keys. The proposed schemes improved the key generation rate, as well as to avoid information leakage during transmission. The proposed physical layer key generation scheme was implemented in a V2V/V2I network system prototype. The extensive experiments in V2I and V2V environments evaluate the efficiency of the proposed key generation scheme. The experiments in real outdoor environments have been conducted. Its key generation rate could exceed 10 bit/s on our V2V/V2I network system prototype and achieve 20 bit/s in some of our experiments. For binary key sequences, all of them pass the suite of statistical tests from National Institute of Standards and Technology (NIST).

## 1. Introduction

In recent years, Vehicle-to-Vehicle (V2V) and Vehicle-to-Infrastructure (V2I) communication bring more and more attention from industry (e.g., Google and Uber) and government (e.g., United States Department of Transportation). In the likely scenario, these Vehicle-to-Everything (V2X) technologies are widely adopted in future autonomous vehicles [1]. In the United States, The National Highway Traffic Safety Administration proposed a mandate in 2016 that would have required all automakers in the United States to install V2V capability by 2020. Although there is not a proposed rule been instated, famous automakers Toyota and Volkswagen declared their intent to deploy V2X technology in the meantime [2]. Vehicles are equipped with more powerful capability in communication [3]. According to the report from the U.S Department of Transportation, it predicted that 60% of all vehicles, the equivalent of a cumulative 146 million cars, will have V2X equipment with the Internet of Vehicles (IoV) technology by 2029 [4]. In China, according to the China Academy of Information and Communications Technology (CAICT)’s white paper, China First Automotive Works (FAW) Group Corporation realized the standard IoV system for all products in 2019. Changan Automobile declared that all new vehicles will be connected to the Internet and equipped with a driving assistance system by 2020. Moreover, all new products will be equipped with human–computer interaction function by 2025. With the development of the V2V/V2I system, both old and new V2V/V2I applications will connect traditional isolated vehicles [5], infrastructure, or anything more through insecure wireless channels. The security issues have already drawn people’s attention [6]. For example, leakage of critical information about the vehicle or the passengers and over the possibility of indirect control of the vehicle’s mechanisms problems mention in reference [7,8,9,10]. V2X technology will eventually cause similar and new security concerns. In V2V/V2I communication environments, there is no public key infrastructure (PKI) and devices communicate on the unauthorized wireless channel. It faced a secure challenge from the broadcast wireless network. Nowadays, a lot of work utilizes the pre-shared key (PSK). However, the PSK is predictably not safe enough. It is not flexible and is easily stolen by attackers. For these reasons, researchers such as those from the European Telecommunications Standards Institute (ETSI) are proposing the following security objectives for V2X communication; confidentiality, integrity, availability, accountability, and authenticity [11].

A major challenge for V2X communication is authentication. Users are exposed to many dangers due to wireless communication. A receiver should verify that a transmitted message was generated by a legitimate user. Current options for securing wireless communications fall under the following types of encryption algorithms; symmetric, asymmetric, and hybrid. Symmetric algorithms are better due to fast performance but they require pre-shared keys. If standardized keys are used, symmetric ways can be predictable and less secure. The asymmetric algorithms allow randomly generated keys but have slow performance. However, V2X connection often handles safety-critical messages with very strict latency requirements, this limitation out all asymmetric and hybrid schemes. For these reasons, secure V2V/V2I communication should face challenges, such as finding a way to generate secret keys for symmetric key algorithms [12].

The physical layer key generation scheme is different from traditional encryption schemes, it is based on the information theory and random characteristics of wireless channels. It is very appropriate for the V2V/V2I system. First, generating a key using the physical layer channel feature is lightweight, which only involves the transmitting and receiving nodes third-party free. Second, the generation and update of the key are performed over the common channel. Third, it is the basis for cross-layer security [13], which provides encryption support for the upper-layer level by generating physical layer keys. Compared to PKI schemes, the physical layer security scheme has no third party and no extra communication overhead. It can provide continuous authentication when PKI is not available. Compared to PSK schemes, the key is generated based on the channel characteristics but not generated based on a fixed value or rule. It also could be updated flexibly according to real-time channel characteristics.

However, there are no appropriate schemes for V2V/V2I communication encryption. The long-distance, mobility, and time-limited nature of V2V/V2I systems pose challenges to the physical layer key generation. The vehicles and infrastructures need to finish communication in seconds and mobile scenarios. The strict latency requirements put forwards challenge to us. In this paper, we first studied the physical layer key generation problem in the V2V/V2I environment. From our previous works [14], we verified the feasibility and suitability of the physical layer key generation on mobile scenarios. Then, we designed a long-range and mobile physical layer key generation scheme for the V2V/V2I system. It utilized the Long Range (LoRa) network to collect the Received Signal Strength Indication (RSSI) as consensus information, it relieved the contradiction on strict latency. Because of the LoRa communication, the preliminary preparation could start kilometers away [15]. It expanded the distance between communication devices to exchange more time for key generation.

The main contributions of this paper are summarized as follows.

- We proposed a long-range and mobile physical layer key generation scheme for V2V/V2I system, which explored the shared randomness extracted from measured RSSI as consensus information to generate secure keys (Section 4).
- We utilized the characteristic of LoRa communication to relieve the contradiction between strict latency and algorithm time overhead in V2V/V2I communication. It allows devices to start signal measurement in kilometers away (Section 4.1).
- We developed the multi-bit quantization algorithm with an improved Cascade key agreement protocol to improve the key generate rate, as well as to reduce the communication overhead and information leakage introduced by the key generation process of V2X devices system communication (Section 4.2, Section 4.3 and Section 4.4).
- We implemented the proposed scheme in a V2X network system prototype and evaluate the key generation efficiency in stationary and mobile scenarios. Experimental results reveal that our proposed scheme can achieve satisfactory key generation performance for upper-layer applications (Section 5).

The rest of the paper is organized as follows. We first survey the related work in Section 2. We introduce the physical layer key generation process and describe the V2V/V2I scenarios in Section 3. The scheme we proposed is described in Section 4 and experimental results are provided in Section 5, respectively. We finally conclude the paper in Section 6.

## 2. Related Work

The physical layer security model is based on Shannon’s communication theory [16]. Wyner describes a wiretap channel model [17]. Wyner’s paper proves that by encoding the content sent by Alice, communication security can be guaranteed without using a communication key. Maurer’s work [18] demonstrates the effectiveness of the Wyner wiretap channel model, points out the incompleteness, and proposes a new scheme to ensure communication security. Most of the research related to the channel parameter key generation is based on Maurer’s work.

Most of the state-of-the-art theories and practical methods for generating secret keys using physical characteristics of the wireless channel (or the physical layer) have been proposed within just the last decade. First, for channel feature extraction, the extracted environment is described in [19,20]. The key negotiation method is systematically described in [21]. As there are some errors in the measurement of the channel parameters, it is necessary to correct the generated initial key, which requires Alice and Bob to negotiate by exchanging error correction information in the public channel. A security enhancement method by discarding secret bit information is provided in [22]. The authors of [23,24] first give the experimental results of the scheme based on the received signal strength to generate the key. In 2019, Mike’s work [25] in sensors seem to avoid error-correcting, but it is not flexible and abandons some of the measurement results. In Jiang’s work [12], it achieves a real outdoor environment experiment and uses the Bluetooth to communicate and collect the RSSI signal. However, Bluetooth transmission distance is only a few hundred meters. For V2V/V2I system, it may not enough. In Xu’s work [26], they explore the LoRa communication for key generation and verify the feasibility of kilometers-away communication. However, they do not propose a real application. The most commonly used channel characteristics include RSSI [12,23,24,25,26], and some works mentioned in [27] used channel state information (CSI) [28,29,30,31,32,33], including channel impulse response (CIR) and channel frequency response (CFR).

However, physical layer security has not been widely applied to the V2V/V2I environment. The communication is characterized by a dynamic environment and high mobility on the communicating entities (vehicles and roadside units) [34]. There are some characteristics in V2V/V2I communication. First, communication happens in diverse environments, e.g., cities, countryside, highway, and so on. Second, there are different communication types, such as vehicle-to-vehicle and vehicle-to-infrastructure. Third, the objects, both static and mobile on the roads, affect communication. The V2V/V2I communication also face security problems. It could be predicted that PKI would be missing in some cases, and the authentication cannot provide continuous services. PSK is a simple solution, but as the key is fixed, it is not safe enough. Another challenge is time-limited. The V2V/V2I devices are time-sensitive, so we have to finish the key generation in limited time.

Facing these challenges, traditional methods are not enough or not entirely feasible, so we consider using a combination of technologies to meet the above challenges. Our work adopts LoRa shield for channel probing. LoRa communication can offer long-range wireless communication for channel probing. Then we adopt a multi-bit quantization algorithm with a level-crossing algorithm to quantify the RSSI signal. It improves the randomness of key generation and the key generation rate. Due to the error codes problem, we adapt and improve the Cascade key agreement protocol [21] to solve it. The improved algorithm effectively reduces the communication overhead during key negotiation. Besides, our proposed key agreement protocol also prevents the disclosure of information. We show a comparison between our work and related works in Table 1. As far as we know, we are the first to adopt LoRa to secure V2V/V2I communication. It expands the communication distance in V2V/V2I communications, and relieve the contradiction on strict latency.

## 3. Key Generation System Overview and V2V/V2I Scenario Description

In this section, we will give an overview of the key generation system and describe the V2V/V2I scenarios. The key generation process can be divided into four steps [27]: channel probing, quantization, information reconciliation, and privacy amplification. In this section, we fully introduce channel measurement and security enhancement. Quantization and key agreement are described in principle here. The specific implementation methods are introduced in Section 4. The V2V/V2I scenarios are complex, there are some vehicles and infrastructures and some of the devices communicate with other devices. Devices broadcast the data package on the unauthorized channel. Some unauthorized nodes also could monitor these data packages.

### 3.1. Channel Probing

**Figure 1:**
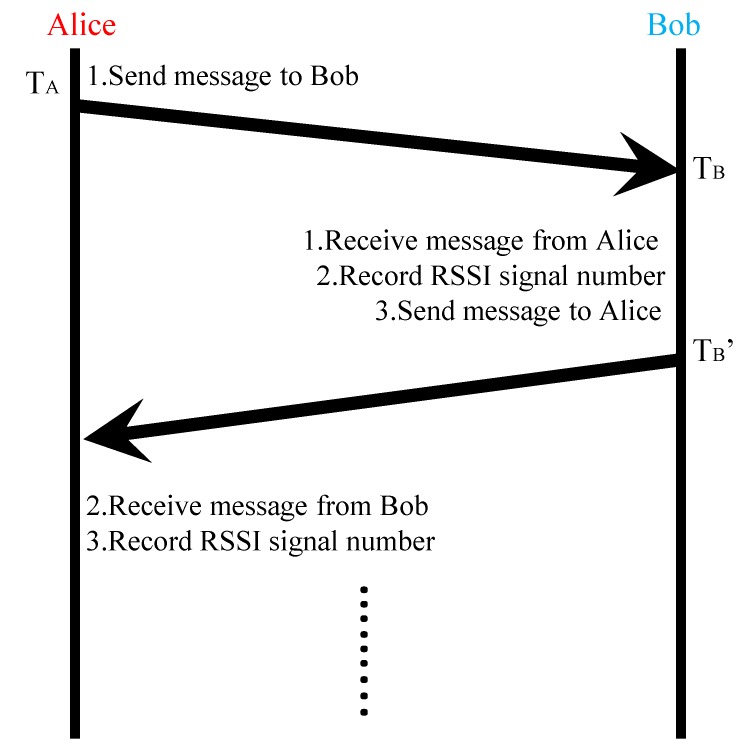
Channel probing process between Alice and Bob.

Channel probing is a key step in obtaining randomness from a channel. It requires two users to alternatively measure the common channel by sending and receiving signals to each other, as shown in Figure 1.

During the channel coherence time, Alice sends a training sequence to Bob at $T_{A}$, and Bob will measure some channel parameters and store them through the received signal. Because of the channel reciprocity of the wireless channel, the channel characteristics at both ends of the link are highly correlated. However, the measured training sequence works in a half-duplex wireless network, Alice and Bob’s measurement time is not synchronous, and the noise will also cause the channel parameters acquired by Alice and Bob to be inconsistent. Our work solves the inconsistent in the follow steps.

Let h(t) be a random function describing the time-varying parameters of the wireless channel between Alice and Bob, assuming that h(t) is the size of the transfer function between Alice and Bob at the fixed test frequency $f_{0}$. Due to the channel reciprocity, the value of the signal transfer function is the same at a given time in the Alice→Bob direction as the Bob→Alice direction. We will represent the channel parameters that need to obtain as *h*, and use h(t) to list the values of the channel parameters at time *t*. To obtain the values of the channel parameters, Alice and Bob should send detection signals to each other to calculate the estimate of h(t), which is recorded as $\hat{h}$. Alice can send a probe signal to Bob after receiving the probe signal sent by Bob, and vice versa. The signals received by Alice and Bob based on continuous detection:

(1) $$r_{a}\left(t_{1}\right)=s\left(t_{1}\right)h\left(t_{1}\right)+n_{a}\left(t_{1}\right),$$

(2) $$r_{b}\left(t_{2}\right)=s\left(t_{2}\right)h\left(t_{2}\right)+n_{b}\left(t_{2}\right),$$

where s(t) represents the sounding signall $n_{a}$ and $n_{b}$ represent the channel noise of Alice and Bob, respectively; and $t_{1}$ and $t_{2}$ are the moments when Alice and Bob receive the sounding signal, respectively. The channel parameters can be calculated separately by Alice and Bob:

(3) $${\hat{h}}_{a}\left(t_{1}\right)=h\left(t_{1}\right)+z_{a}\left(t_{1}\right),$$

(4) $${\hat{h}}_{b}\left(t_{2}\right)=h\left(t_{2}\right)+z_{b}\left(t_{2}\right),$$

where $z_{a}$ and $z_{b}$ are the values obtained by processing the channel noises $n_{a}$ and $n_{b}$ by the estimation function, respectively, ${\hat{h}}_{a}$ and ${\hat{h}}_{b}$ are almost completely unequal. This is because there is channel noise estimation error and reception lag time $\tau =|t_{2}-t_{1}|$. By repeatedly transmitting the sounding signals in an alternating manner on the time-varying channel, Alice and Bob can generate a pair of highly correlated sequences containing *n* estimates:

$${\underline{\hat{h}}}_{a}=\left\{{\hat{h}}_{a}\left[1\right],{\hat{h}}_{a}\left[2\right],\cdots ,{\hat{h}}_{a}\left[n\right]\right\}$$

$${\underline{\hat{h}}}_{b}=\left\{{\hat{h}}_{b}\left[1\right],{\hat{h}}_{b}\left[2\right],\cdots ,{\hat{h}}_{b}\left[n\right]\right\}$$

**Figure 2:**
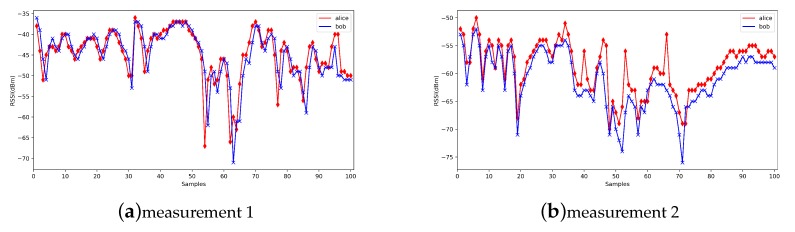
Received Signal Strength Indication (RSSI) measurements from previous works.

We show some previous RSSI measurements here (Figure 2). From previous measurements, we find that in mobile scenarios, the measurements are fluctuant and suitable for the physical layer key generation. Jana’s work [24] and Xu’s work [26] give the detailed mathematical analysis on RSSI signals. The signal strength would be influenced by the environments, distance and moving speed, but the signal strength between Alice and Bob is still reciprocal and high correlated. It is suitable for the key generation.

For Eve, the eavesdropper can eavesdrop information from Alice and Bob, but the signal it receives is completely different from what the legitimate parties receive. If the distance is greater than λ/2 between Eve and Alice or Eve and Bob, then Eve and Alice and Bob’s channel parameters $h_{ae}$ and $h_{be}$ are not related to *h*. Therefore, Eve cannot calculate the corresponding channel parameters.

### 3.2. Quantization

When Alice and Bob exchange multiple packets, each packet establishes a time sequence for measuring RSSI. Then, based on its time sequence to generate an initial secret bit sequence. The measured values map to binary values, specifically, the quantization, is done based on the specified threshold. Different quantizers have proposed in existing papers. These quantizers differ primarily in their choice of thresholds and in the number of thresholds they use [35].

There are two main problems with quantization. First, hardware noise, ambient noise, and measurement errors can affect the results of RSSI measurements. Second, in the V2X devices network system, the RSSI values collected over a while are inevitably correlated in one RSSI signal sequence. To solve these problems, we propose a multi-bit quantization algorithm. The adaptive multi-bit quantization algorithm used in this paper describes in detail in Section 4.

### 3.3. Information Reconciliation

After completing the first two steps, Alice and Bob use the quantizer to quantize the original channel parameter information obtained by the channel measurement to obtain a bit sequence. The quantized results at this time cannot be directly used as keys because there are different bits in their bit sequence. The reasons are manifold, mainly including channel noise and interference, hardware device differences, and Alice and Bob cannot simultaneously collect data due to the half-duplex working mode of the wireless network.

Information reconciliation corrects the inconsistent bits under the premise of providing less interactive information about the key content. The Cascade key negotiation algorithm [21] is widely adopted in many past studies in the physical layer key generation. However, the original Cascade has too much communication overhead and leak information between key agreements. In this paper, we implement the improved Cascade protocol as our experimental scheme. It already verifies effectiveness in our previous work [14].

### 3.4. Privacy Amplification

Privacy amplification mainly solves two problems: the first is that in theory, we can only detect the channel once in the coherent period so that the channel measurement results are guaranteed to be independent. However, in actual situations, due to environmental changes and other factors that cause unpredictable channel changes, it is very difficult to estimate the coherence time of the channel. In the course of channel measurement, as two corresponding measurements occur within the coherence time, one bit and subsequent bits may be correlated. Therefore, the bit sequence obtained from the channel change can exhibit a short-term correlation between subsequent bits. We need a mechanism to minimize the correlation between bits in the bit sequence and enhance the randomness of the key. The second is that we need a mechanism to eliminate some of the bits that interact during key negotiation so that the opponent cannot use this information to guess the portion of the extracted key.

The privacy amplification is to process the data after key negotiation through a one-way hash function to improve the security of the key. This paper does not discuss privacy amplification.

### 3.5. V2V/V2I Scenario Description

In the V2V/V2I scenarios, there are a lot of vehicles and infrastructures. In V2V/V2I scenarios, vehicles could communicate with other vehicles. The communication initiator is considered as Alice, and another is considered as Bob. Alice and Bob are communicating on the unauthorized channels. Alice and Bob could start channel probing from kilometers away, after channel probing, Alice and Bob could finish the quantization without each other. When Alice and Bob come close, they could finish the information reconciliation, it could help Alice and Bob start the key generation kilometers away. It uses spatial distance to alleviate the time-limited problem.

However, there are some attacker Eve. We assume that Eve only listens to all of the communication between Alice and Bob. Eve can distinguish the message’s sender, and then measure the channel signal characteristics for key generation. Eve also knows the key generation algorithm and the value of the parameters used in the algorithm. However, Eve cannot be very close to either Alice or Bob (about half of wavelength), which ensures that Eve has difficulty measuring a correlated signal sequence. Eve does not jam the communication and modify the message exchanged between Alice and Bob. In short, Eve is not interested in disrupting the key establishment between Alice and Bob. We also assume that Eve does not cause a person-in-the-middle attack. In other words, our proposed scheme works against passive adversaries.

**Figure 3:**
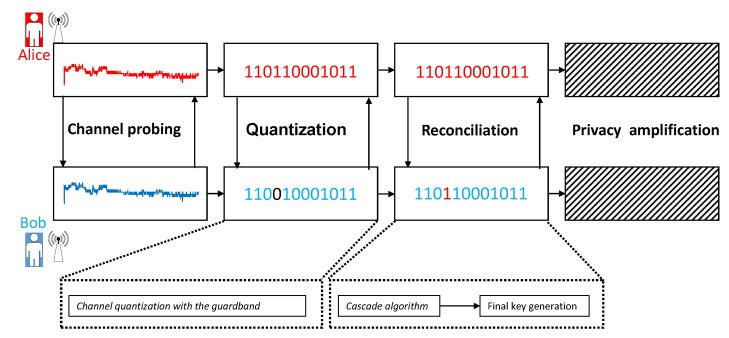
Physical layer key generation process in our proposed scheme.

We show our scheme’s work-flow in Figure 3. We adopt RSSI signals as physical layer key parameters in our work. The channel probing works as above. The main contribution of our work is quantization and reconciliation. The quantization in our work has two steps. First, we use an adaptive lossy quantizer to transfer RSSI signals to a binary sequence. Second, the level-crossing algorithm is used to generate the initial key sequences. Then we propose an information-hided Cascade algorithm to generate the final key. After these steps, we can get a usable secret key between Alice and Bob.

## 4. LoRa Based V2V/V2I Key Generation Scheme

**Figure 4:**
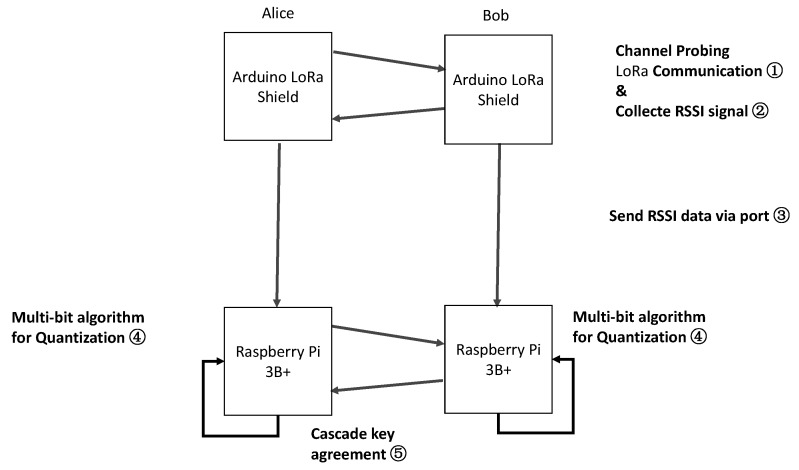
LoRa-based V2V/V2I key generation system model.

In this section, we describe our LoRa-based V2V/V2I key generation scheme in detail. Our system model has two main parts: the channel probing part and calculation part. The channel probing part could finish signal measurement in long-distance away. After the signal measurement, the channel probing part sends the RSSI sequence to the calculation part, the calculation part will finish the quantization and information reconciliation. In previous work [36], we are inspired by the simultaneous use of multiple communication technologies. In future work, the calculation part will also finish the privacy amplification. As far as we know, we are the first to adopt LoRa to secure V2V/V2I communication. We show our system model in Figure 4.

### 4.1. Channel Probing

As we discussed in the last section, the first step of the key generation is channel probing. However, for the V2X system, the communication party may not have enough time to send a channel probing package. This is why, in Jiang’s work [12], Bluetooth is not enough for the V2X system. In our LoRa-based V2X key generation scheme, the LoRa shield could measure RSSI between long-range places. Therefore, the communication part could begin the channel probing in advance. Using the LoRa shield helps the V2X devices utilize the spatial distance to alleviate the contradiction of time. In Xu’s work [26], the maximum achievable distance is ~5 km.

### 4.2. Signal Quantization

After channel probing, both Alice and Bob get their RSSI sequences. In the V2X wireless network, the RSSI values collect over a while and are inevitably correlated in one RSSI signal sequence. Therefore, we need an algorithm to reduce the correlation. For these reasons, we adopt the level-crossing algorithm [25]. It reduces the correlation between RSSI signals.

The multi-bit quantization algorithm is based on the single-bit quantization algorithm. In previous work [37], we adopt the single-bit quantization algorithm. The channel parameter sequence obtained by channel measurement is a continuous random number. To convert it into a bit sequence, the most critical part is the choice of the quantizer. The choice of the quantizer is related to the efficiency of the quantization algorithm. It uses a double threshold quantizer:

(5) $$Q\left(x\right)=\left\{\begin{array}{ccc} 1 & \; & x>q_{+}\\ 0 & \; & x<q_{-} \end{array}\right.,$$

**Figure 5:**
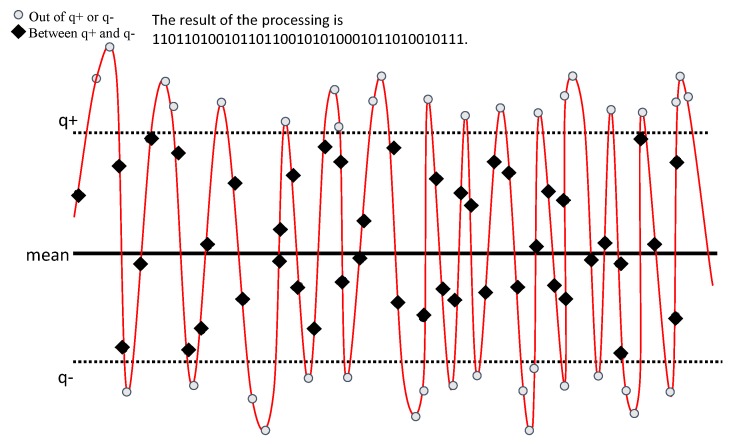
Single-bit quantization schematic diagram.

Take Figure 5 as an example, we can see that the quantizer works in the following way; the original channel parameter sequence is processed by the quantizer, and a bit sequence can be obtained.

The choice of the two thresholds $q_{+}$ and $q_{-}$ is calculated based on the statistical information obtained from the channel measurement. The channel parameter changes are by the Gaussian distribution, and the threshold is selected as follows,

(6) $$q_{+}=mean+\alpha \cdot \sigma ,$$

(7) $$q_{-}=mean-\alpha \cdot \sigma ,$$

where mean refers to the average of the channel parameters, α is the custom parameter, and σ is the variance of the channel parameters.

**Figure 6:**
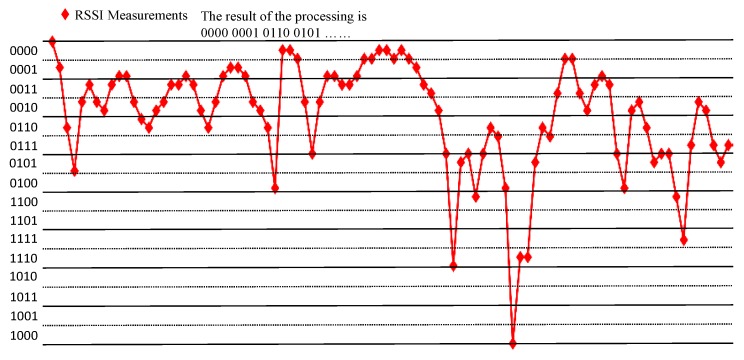
Multi-bit quantization schematic diagram.

The single-bit quantization algorithm reduces the key achievable rate and key generation rate because it wastes a lot of effective information from the RSSI signal sequence. The time-limited on V2V/V2I communication request us to design a fast quantizer, so we propose multi-bit quantization. It employs all of the RSSI signals, and quantifies the single signal to four binary bits, improves the key generation rate, and decreases the communication time. However, if we use all of the RSSI signals, it causes difficulty to error correct. To solve this problem, we adopt the Gray code to mitigate it. From our past experience. The range between the RSSI sequences is over 16. The algorithm describes as Algorithm 1, and we give an example after the pseudo-code in Figure 6.
**Algorithm 1**: Multi-bit quantization algorithm.**Require:** List *R* of RSSI signal measurements.**Ensure:** initial key sequence $K_{1}$Range = Max(*R*) − Min(*R*)interval = Range ÷ 16$K_{1}$ = blank_string**for**i=1tolength(R)**do** **for** j=0 to 15 **do**  **if** (R[i] > Min(*R*) + *j* * interval) & (R[i] <= Min(*R*) + (j+1) * interval) **then**   temp← Graycode(j)   $K_{1}$ += temp
  **end if** **end for****end for**


For more V2X scenarios, the environments are complex and uncertain, we could also consider designing an adapted quantization algorithm to face the complex and uncertain environments in future works.

### 4.3. Level-Crossing Algorithm

**Figure 7:**
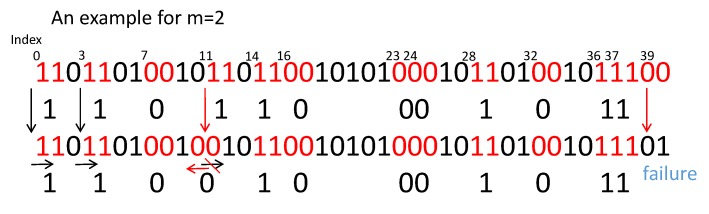
Level-crossing algorithm.

Now, as discussed above, we adopt the level-crossing algorithm [25]. We show an example as Figure 7. Assume that when the algorithm is running, Alice and Bob collect enough channel estimates and by alternatively detecting the channel between them. Assume that the channel estimates and have the same length.

The pseudo code is shown as Algorithm 2.**Algorithm 2**: Level-crossing algorithm.**Require:**${\hat{\underline{h}}}_{a}$ and ${\hat{\underline{h}}}_{b}$**Ensure:** Alice and Bob’s $K_{a}$ and $K_{b}$ Alice:**for**i=1to$length({\hat{\underline{h}}}_{a})-m$**do** **if** $Q\left({\hat{h}}_{a}\left[i\right]\right)=Q\left({\hat{h}}_{a}\left[i+1\right]\right)\cdots =Q\left(\left({\hat{h}}_{a}\left[i+m-1\right]\right)\right)$ **then**  $i_{end}\leftarrow last\;\;index\;\;in\;\;excursion$
  $L^{\prime }\leftarrow \left[L^{\prime }\;\;;\left\lfloor \frac{i+i_{end}}{2}\right\rfloor \right]$
  $i\leftarrow i_{end}+1$
 **else**  i←i+1
 **end if****end for**$L=Random\;\;subset\;\;of\;\;L^{\prime }$Alice send L to Bob PUBLIC_CHANNEL Bob:**for**l∈L**do** **if** $Q\left({\hat{h}}_{b}\left[l-\left\lfloor \frac{m-2}{2}\right\rfloor \right]\right)=\cdots =Q\left({\hat{h}}_{b}\left[l+\left\lceil \frac{m-2}{2}\right\rceil \right]\right)$ **then**  $\tilde{L}\leftarrow \left[\tilde{L};\;\;l\right]$
 **end if****end for**$K_{b}=Q\left({\hat{h}}_{b}\left(\tilde{L}\right)\right)$Bob send $\tilde{L}$ to Alice on PUBLIC_CHANNEL Alice: $K_{a}=Q\left({\hat{h}}_{a}\left(\tilde{L}\right)\right)$


After completing the above steps, as in the previous work [14], Alice and Bob use information reconciliation to correct the mismatching bits to the reconciled bitstream and extract a high entropy bitstream.

### 4.4. Lightweight Key Agreement Protocol

**Figure 8:**
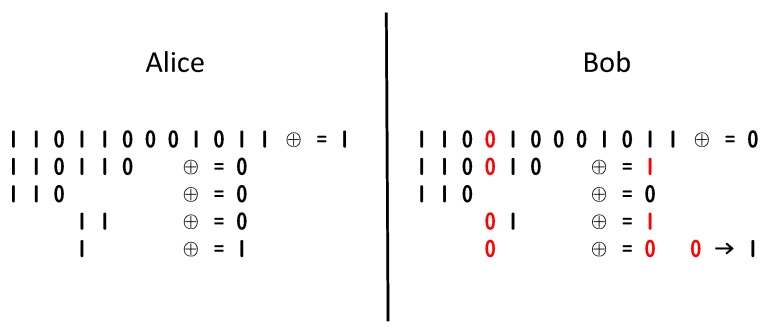
Binary error correction algorithm.

As discussed in Section 3, The legitimate communication parties Alice and Bob obtain an initial sequence of the same length and with certain inconsistent bits. To correct the inconsistent bits, Alice and Bob are required to perform the exchange of error bit related information on the common channel. Cascade protocol [11] is a widely adopted algorithm in past studies. The original Cascade protocol has some problems, such as too many interactions and the last negotiation leaks complete information. We improve it to reduce these problems. The Cascade algorithm is based on the binary error correction algorithm. We show an example in Figure 8.

The Cascade algorithm just repeated uses binary error correction.

1. It groups the sequence by M bits. For every group, it uses binary error correction.
2. It repeats the first step for several rounds and shuffles the sequence after every round.
3. Then backs sort from last round to first round to find other error.

Although the above algorithm has highly correction efficiency, the disadvantages are also obvious. Assuming that there are odd error bits, the group length is *k*, so the error correction process through the dichotomy requires $log_{2}k$ interactions. In the back-tracking process of the i−th round, multiple groups need to be continuously corrected, so when the group length is large, Alice and Bob should change much information during the key negotiation process. There will be a lot of interactions, which will have an impact on the efficiency of the key agreement. In addition, when the search range of the error bit is locked at 3 bits or less, if the error correction using the binary error correction algorithm is continued, the corresponding bit information will be completely leaked during the search process.

**Figure 9:**
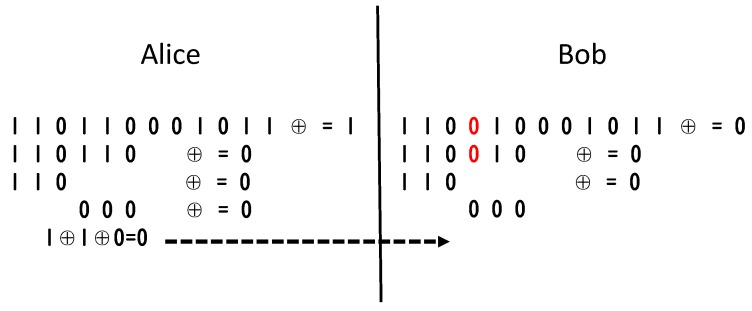
Information-hided cascade algorithm.

Therefore, in this paper, we propose an improved scheme as Figure 9. When the binary error correction search range is reduced to less than 3, the error correction is not used, and the bit of this part is filled as the exclusive result of Alice. The position of the three bits is recorded, and the bits are deleted after the backtracking is finished. In this way, our algorithm will not affect the subsequent iterative backtracking error correction on the basis of preventing information leakage. It also reduces the number of Alice and Bob’s information interactions, which can improve the efficiency of key negotiation.

## 5. Performance Evaluation

In this section, we conduct extensive experiments and evaluate the performance of our proposed scheme. First, we introduce the V2X network system prototype and present the evaluation metrics for the experiments. Second, we conduct extensive experiments in real indoor environments to evaluate the performance based on the evaluation metrics. Third, we analyze the security performance of the proposed scheme against an eavesdropper.

### 5.1. Experiment Set-Up and Evaluation Metrics

**Figure 10:**
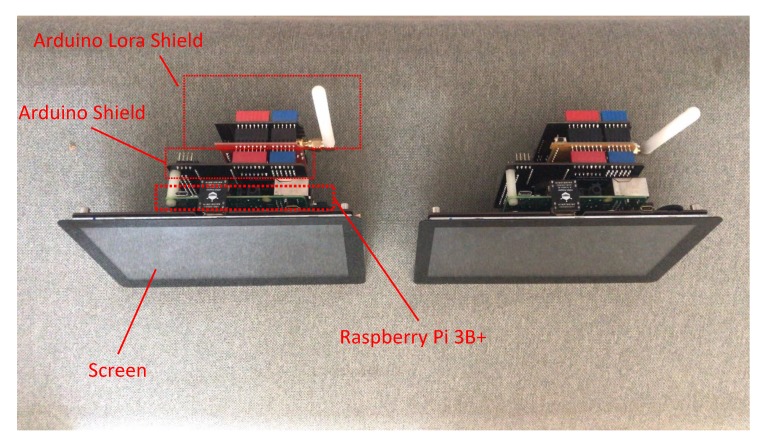
The devices used to build the key generation system.

Our evaluation environment includes two LoRa shields with Raspberry Pi 3B+ with the operating system Raspbian and a Raspberry Arduino expansion boards. The LoRa Shield and WIFI Shield are LoRa expansion boards that can be mounted on the Arduino Leonardo board using the SX1278 chip. Two of these platforms are set up as Alice and Bob. The experimental environment is shown in Figure 10. The experiment involves Alice sending a request message to Bob, which then replies with a response. The request and response packet may be lost. In this case, Alice will record the last RSSI signal.

**Figure 11:**
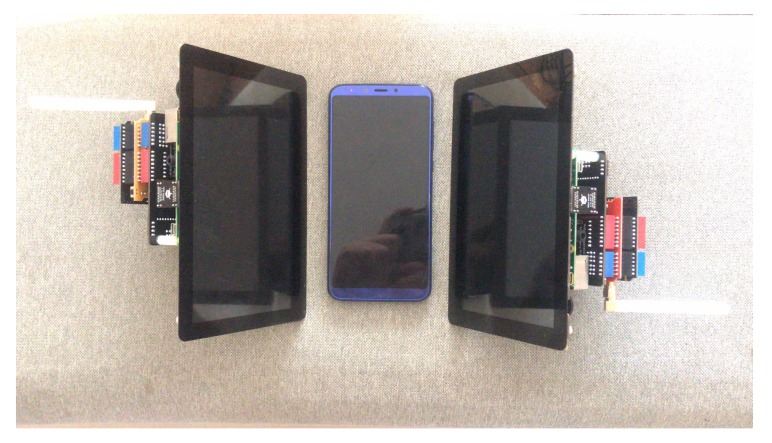
The size of communication nodes compare with normal smart phone.

The size of the screen is approximately 9 inches; it is like the size of the car’s computer size. We compare the whole devices with a normal smartphone in Figure 11.

For our proposed distributed physical layer key generation scheme, we focus on the following evaluation metrics:

1. Achievable key ratio: It represents the ratio of generated key to the collected RSSI signals.

2. Key generation rate: It denotes the average number of key bits that can be generated per second.

3. Entropy: It is the measure of uncertainty or randomness associated with the generated key. The entropy of a binary key varies in the range [0,1], and larger entropy indicates more randomness of the key. In our work, we apply the NIST [38] suite of statistical tests to evaluate the randomness of the generated key.

**Figure 12:**
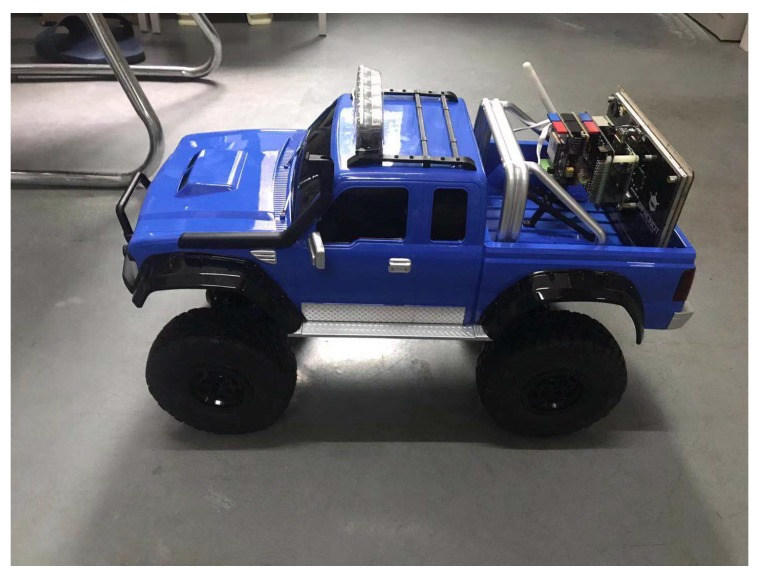
The model car carries the communication node.

We use some model cars (Figure 12) to carry the communication nodes, the communication nodes display at the back of the model car. The car’s speed could up to 25 km/h (~7 m/s). We use two of these model cars to simulate the real car movement.

Experiments are conducted in four outdoor environments. Under each environment, we collect packages from Alice and Bob. The outdoor experiments are conducted on a normal road with some cars and buildings. Environments list in the following figures.

**Figure 13:**
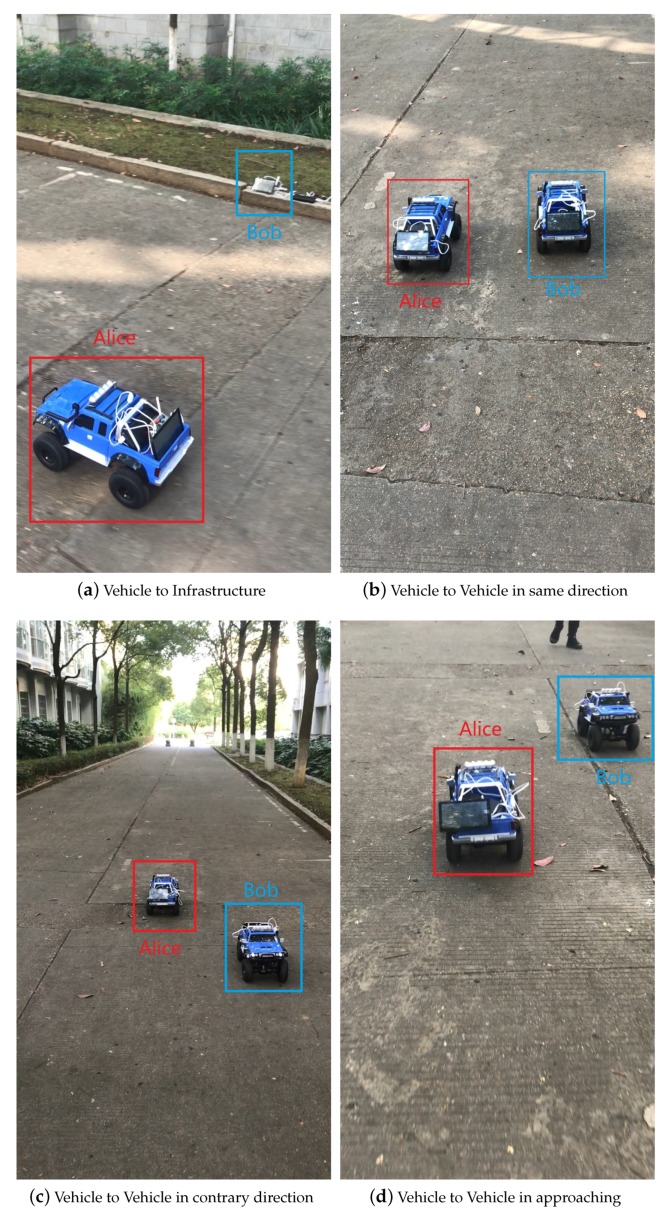
Four experiment environments.

- Experiments 1 Vehicle to Infrastructure. (Figure 13a)
- Experiments 2 Vehicle to Vehicle in same direction. (Figure 13b)
- Experiments 3 Vehicle to Vehicle in contrary direction. (Figure 13c)
- Experiments 4 Vehicle to Vehicle in approaching. (Figure 13d)

We summarized the main experimental results in Table 2, including the range between max signal strength and min signal strength, signal correlation between Alice and Bob, and the key generation rate in different experiments.

### 5.2. Key Generation Results

**Figure 14:**
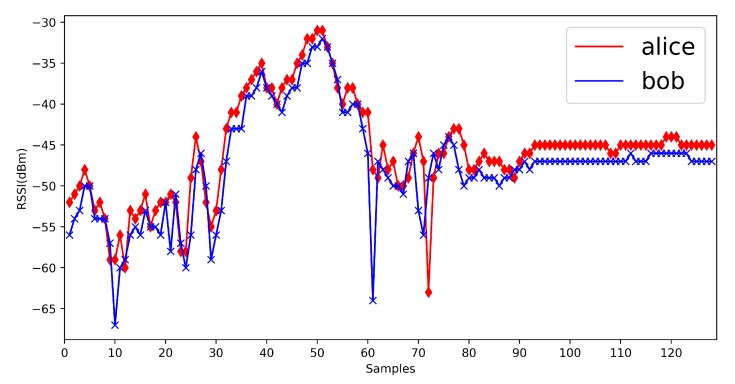
Vehicle to infrastructure.

**Figure 15:**
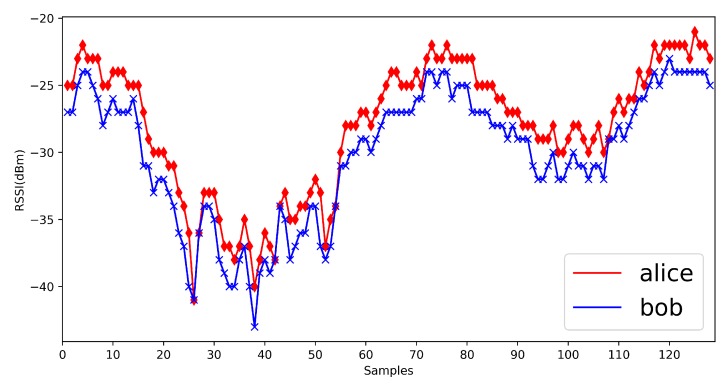
Vehicle to vehicle in same direction.

**Figure 16:**
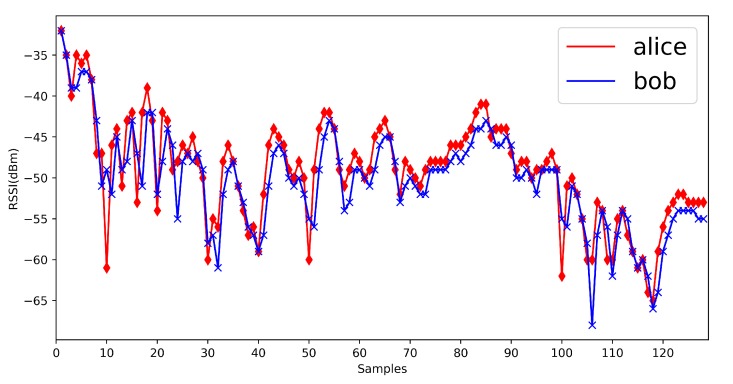
Vehicle to vehicle in contrary direction.

**Figure 17:**
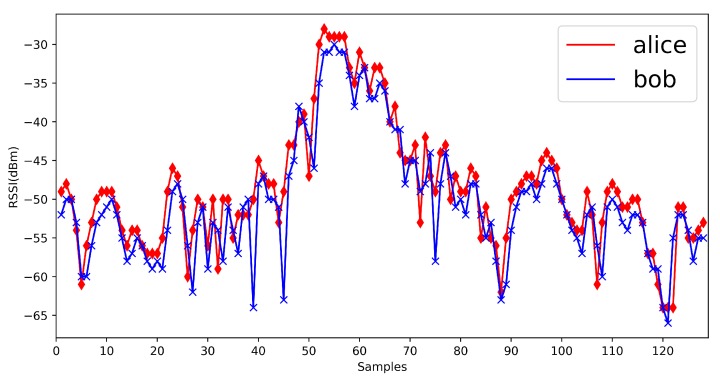
Vehicle to vehicle in approaching scenario.

First, we show the RSSI measurements in four environments (Figure 14, Figure 15, Figure 16 and Figure 17). The range of RSSI signals in four environments is all over 16. For these results, the quantization algorithm is suitable for our V2X key generation scheme.

Then, we discuss the achievable key ratio and the key generation rate. In our quantization algorithm, all of the RSSI signals are quantified, and none of the signals are discarded. Therefore, our achievable key ratio is 100%. For every single RSSI signal, it could be quantified to 4 binary bits. Therefore, our key generation rate is over 10 bits/s, and sometimes up to 20 bits/s. We can generate a 512 bits binary sequence from 19 s to 40 s.

From the measurement results, we can know the physical layer key generation is suitable for the V2X environment, and our V2X key generation scheme is feasibility and high efficiency.

### 5.3. Key Entropy and Randomness

To evaluate the randomness of the generated key, we apply the NIST [38] suite of statistical tests after our key generation. The NIST statistical test gives the *p*-values of different random test processes, and the *p*-values indicate the probability that the key sequence is generated by a random process. Conventionally, if the *p*-value is less than 1%, the randomness hypothesis is rejected which means the key is not random. In Table 3, we show the result of the NIST statistical test.

### 5.4. Security Analysis

**Figure 18:**
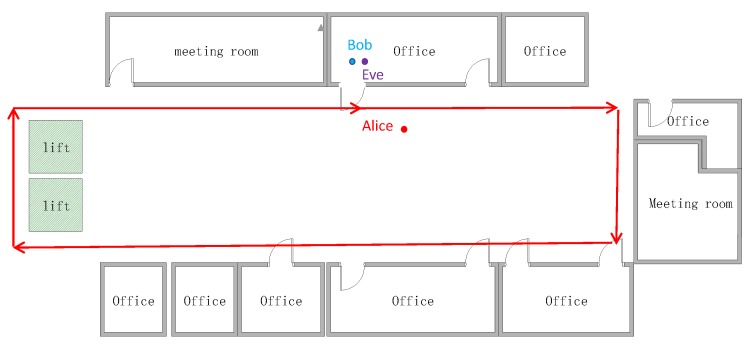
Mobile scenario and the moving trace.

**Figure 19:**
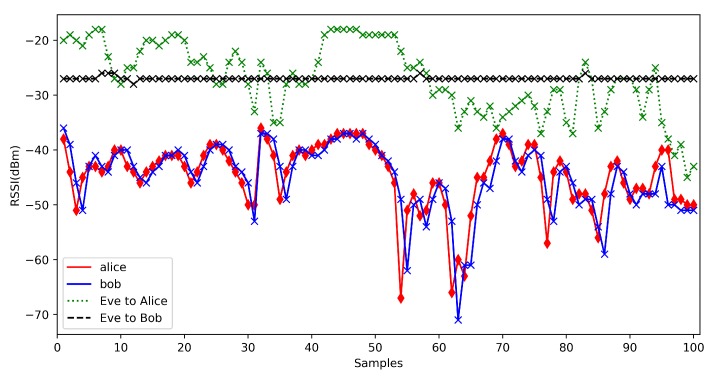
RSSI of Alice Bob and Eve with mobile scenarios.

As the RSSI signals collect at different times are different. Eve is hard to collect the same RSSI signals that Alice receives or Bob receives. However, Eve can measure RSSI signals. From our result, we know that the RSSI measured by Alice and Bob are highly correlated, whereas the RSSI between Eve and Alice/Bob is significantly less correlated. For our previous work [14], we tested the correlation between Eve and Alice or Bob. The test trace shows in Figure 18 and the result shows in Figure 19. Eve is hard to calculate the final key between Alice and Bob. Algorithm 2 also tells us that Alice and Bob only send the index of signal sequence on the public channel, Eve is not easy to get the same sequence index in its signals sequence. Moreover, if Eve gets the same index numbers, its signal sequence is different from Alice’s and Bob’s, and it can not calculate the same initial binary bit sequence from the index and its signal sequence. After quantization, Alice and Bob use the Information-Hided Cascade algorithm, the initial bit sequence may change. For these reasons, the possibility of stealing a key is very low.

### 5.5. Discussions

From the above experiments, we verify the feasibility and high efficiency of our V2X key generation scheme. The LoRa shield helps us solve the problem of limited time because it can measure the channel characteristics from long distance. The multi-bit quantization algorithm helps us increase the achievable key ratio and key generation rate. The level-crossing algorithm helps us improve entropy. In the next step, we can try to find other channel characteristics for channel probing.

In our real-world outdoor experiments, we also face some problems. The power of the power bank is not stable. Sometimes, the Raspberry Pi will reboot when the model car is running. We think we should reduce the power of our experiment devices, or adopt large power, which is larger than 20 W. The model car is hard to control, and could not set a stable speed. In future works, we could try to employ an automatic model car with tracks, or some real cars to conduct our experiment.

In the real car environments, the speed is higher, the environments are more complex, and the communication distance will be several kilometers. For example, the speed is ~40–60 km/h. The communication quality may become worse since the signal strength is down to −70 to −80. In the real velocities experiments, we may face more challenges. In [24], the authors conducted experiments in complex environments. However, even the speed is higher, and the strength is worse, the signal between Alice and Bob is still reciprocal and high correlated. It still could generate the same key according to the correlated signals.

With the development of the V2X technology and the large-scale deployment of V2X node, the physical layer key distribution technology has broad prospects. However, the next to do is to reduce the memory footprint based on existing algorithms and improve algorithmic efficiency, enabling large-scale deployment on compute and storage capable endpoints. The key generated by the physical layer channel feature can be used as the encryption key of the upper layer protocol, and other work could be developed on the existing basis.

## 6. Conclusions and Future Work

This paper implements a distributed and lightweight physical layer key generation scheme for secure V2V/V2I communication. We adopt LoRa communication for channel probing; it helps us measure signal sequence in advance. The multi-bit quantizer and the level-crossing algorithm generate the initial key sequences and improve the key generation rate. The information-hided Cascade algorithm [11] effectively reduces the number of information interactions based on the original algorithm. Extensive experiments show that our scheme can generate the same key for two V2V/V2I devices under different environments. Our key generation scheme reaches 100% on achievable key ratio and our key generation rate exceeds 10 bit/s. The key has not been used to up-layer. In the future, we will try to make a whole protocol system for the V2X system. We would try to design a whole system from low-layer key generation to up-layer protocols. This is the focus of our future work. We will also try to find better quantization algorithms to increase the key generation rate, and continue to improve the randomness of our key. Additionally, as the current off-the-shelf LoRa nodes can only provide RSSI information, exploring more channel characteristics such as Channel Impulse Response (CIR) to accelerate signal acquisition is also an interesting research direction. 

## Figures and Tables

**Table 1:** The comparison between our work and related works.

Works	Technique	Platform	Distance	V2V/V2I Scenarios
[12]	Bluetooth	Bluetooth Dongles	short	Yes
[25]	802.11n	TL-WN722N	short	No
[26]	LoRa	LoRa Shield	long	No
Our LoRa-based work	LoRa	Arduino LoRa Shield	long	Yes

**Table 2:** Main Outdoor experimental results.

Environment	Exp.1	Exp.2	Exp.3	Exp.4
Max Signal Strength	−31	−21	−32	−28
Min Signal Strength	−67	−43	−68	−66
Range	36	22	36	38
Signal Correlation	0.908	0.986	0.902	0.921
Key Generation rate	16.5 bit/s	26.9 bit/s	22.2 bit/s	12.8 bit/s

**Table 3:** *p*-values of NIST statistical test.

Environment	Exp.1	Exp.2	Exp.3	Exp.4
Frequency	0.859	0.859	0.288	0.723
Block Frequency	0.157	0.077	0.271	0.181
Cumulative sums(Fwd)	0.997	0.067	0.265	0.737
Cumulative sums (Rev)	0.999	0.103	0.574	0.574
Runs	0.773	0.621	0.351	0.360
longest run of ones	0.077	0.808	0.763	0.759
FFT	0.871	0.144	0.871	0.516
Approx. Entropy	0.048	0.392	0.602	0.492
Serial	0.038 & 0.033	0.924 & 0.723	0.606 & 0.479	0.535 & 0.288
